# A brief neuromotor-therapy-augmented pathway for pediatric functional movement disorders: a sequential historical cohort study

**DOI:** 10.3389/fneur.2026.1968237

**Published:** 2026-10-30

**Authors:** Baglioni Valentina, Notaristefano Ilaria, Maggiore Patrizia, Romano Sara, Galosi Serena, Tinazzi Michele, Geroin Christian, Esposito Dario

**Affiliations:** 1 Department of Human Neuroscience, Sapienza University of Rome, Rome, Italy; 2 Department of Neurosciences, Biomedicine and Movement Sciences, University of Verona, Verona, Italy; 3 Department of Surgery, Dentistry, Pediatrics and Gynecology, University of Verona, Verona, Italy; 4 Neurology Unit, Movement Disorders Division, Department of Neurosciences, Biomedicine and Movement Sciences, University of Verona, Verona, Italy

**Keywords:** functional neurological disorder, historical cohort study, motor rehabilitation, pediatric functional movement disorders, pediatric neurorehabilitation

## Abstract

**Background:**

Pediatric functional movement disorders (FMD) often improve, but access to specialized motor rehabilitation is heterogeneous. We compared two sequential, non-overlapping care periods within one clinical unit: usual care (UC) and a neuromotor-therapy-augmented pathway (NMT-A), which added brief hospital-based neuromotor therapy before community referral.

**Methods:**

This retrospective, non-randomized historical cohort included 17 UC patients (June 2021–January 2023) and 29 NMT-A patients (February 2023–June 2025). The primary outcome was continuous S-FMDRS at 6 months; S-FMDRS-defined motor symptom-free status (score = 0) was secondary. Both were reassessed at 12 months, together with community-treatment engagement.

**Results:**

At 6 months, the adjusted NMT-A-minus-UC S-FMDRS difference was −1.66 points (95% CI, −4.04 to 0.72). Motor symptom-free status occurred in 55.2% versus 5.9% (risk difference, 49.3 percentage points; 95% CI, 21.8 to 66.4), and in 86.2% versus 52.9% at 12 months (risk difference, 33.3 percentage points; 95% CI, 6.5 to 56.8). The pathway-by-time interaction was not significant. Sustained community physical and/or occupational therapy was documented in 75.0% versus 17.6% (RR, 4.25; 95% CI, 1.74–12.46).

**Conclusion:**

The NMT-A pathway-period was associated with more frequent motor symptom-free status and greater continuity of community motor rehabilitation, although the adjusted mean S-FMDRS difference was not statistically significant. Because pathway and calendar period were inseparable, these findings should be interpreted as associations with the overall later care pathway rather than as an isolated effect of NMT.

## 1 Introduction

Functional Movement Disorders (FMD), a subtype of Functional Neurological Disorders (FND), encompass weakness, tremor, dystonia, and other abnormal movement patterns that meet positive diagnostic criteria of inconsistency and incongruence relative to other recognized neurological disorders (1–3). Symptoms arise from dysfunctional interactions among networks involved in motor control, emotion regulation, attention, and sense of agency rather than from structural lesions (1, 4, 5). Impaired predictive coding may generate movements experienced as involuntary; neuroimaging studies have also shown reduced activation of motor and supplementary motor areas with abnormal limbic connectivity (6–10). These mechanisms support a biopsychosocial, multidisciplinary approach, particularly in pediatrics, where family involvement and psychosocial assessment are essential (6, 11, 12). These mechanistic considerations have direct clinical relevance in childhood, where frequency, prognosis, and access to care shape treatment needs. In childhood and adolescence, FND accounts for up to 10% of presentations to general pediatric neurology clinics and up to 20% in pediatric epilepsy clinics, although estimates vary by setting and case definition (13). Prognosis is generally favorable, and many children achieve symptom resolution when diagnosis and treatment are timely (14–16). Even when eventual recovery occurs, a prolonged course can disrupt school attendance, physical activity, peer participation, and family routines; time to remission may therefore be clinically relevant in addition to final symptom status (14–17). Access to appropriate and specialized care remains limited. Diagnostic delays of up to 24 months from symptom onset have been reported, increasing the risk of symptom consolidation and chronicity (18, 19). Physical therapy is now recognized as a cornerstone of FMD treatment, particularly through motor retraining aimed at restoring automatic movement patterns. Yet access to specialized FMD-informed pediatric rehabilitation remains limited and highly variable. Children are often managed through psychoeducation, psychological referral, community-based therapy, and aftercare recommendations; access to clinicians with specific FND rehabilitation expertise remains inconsistent (20). Most structured protocols were developed in adult populations, and pediatric-specific pathways remain scarce. Within these care gaps, positive diagnostic communication and psychoeducation provide the necessary foundation for treatment, but they must connect children and families to effective rehabilitation. Explaining the diagnosis through positive clinical signs, providing a coherent account of symptom mechanisms, and emphasizing the potential for reversibility can support acceptance of the diagnosis and engagement with treatment (6, 19, 20). Their clinical value depends on whether children and families can translate this understanding into functional practice and obtain appropriate rehabilitation. When community clinicians have limited FND-specific experience, the transition from tertiary diagnosis to local treatment may become a point at which continuity and treatment coherence are lost (20). The present study compared two successive care configurations that differed specifically in how this transition was managed. The usual care (UC) group consisted of diagnostic communication, psychoeducation for the child and family, and referral to community-based rehabilitation or psychological services without an initial specialist rehabilitation phase. In the neuromotor-therapy-augmented (NMT-A) group, these same components were retained but were preceded by a brief specialist-delivered neuromotor intervention designed to initiate FMD-specific motor relearning and prepare subsequent community-based treatment. That transition from diagnostic understanding to functional practice places rehabilitation design at the center of pediatric FMD care. Rehabilitation for FMD typically focuses on restoring automatic movement through motor retraining, often combined with psychological intervention. Neuromotor approaches integrate attentional, motor-learning, imagery, and sensory-based strategies to improve motor competence and recalibrate body representation (21–27). Pediatric programs have reported high rates of symptom resolution (12, 14, 16, 22, 24). Many require intensive multidisciplinary or inpatient resources and remain difficult to scale. A model in which a specialist team initiates FMD-informed motor relearning and prepares subsequent treatment by community providers may offer a lower-intensity alternative when prolonged specialist rehabilitation is not locally available. Children with FMD also frequently present with attentional, emotional, and neurodevelopmental vulnerabilities (28, 29), supporting rehabilitation adapted to developmental level, learning style, and family context. The need for a developmentally adapted, lower-intensity model provides the rationale for the care configuration evaluated here. Published rehabilitation models have primarily centered on physical therapists and occupational therapists (21, 22, 26). In the Italian clinical unit studied here, comparable FMD-informed principles were implemented by a healthcare professional specialized in developmental-age motor and psychomotor rehabilitation, the Neuro and Psychomotor Therapist of Developmental Age (TNPEE), whose developmentally oriented framework combines sensorimotor and functional training with relational, family-centered, and play-based strategies (30–32). This configuration supported evaluation of a brief, developmentally informed rehabilitation pathway within a public health system. Against this background, we used a retrospective, non-randomized, real-world historical cohort design to compare motor outcomes across two sequential, non-overlapping care periods within one clinical unit at a single tertiary pediatric hospital. The primary outcome was 6-month motor severity measured continuously with the S-FMDRS; motor symptom-free status at 6 months was a secondary exploratory outcome. Both outcomes were reassessed at 12 months to characterize the subsequent motor course, and sustained engagement with community-based treatments was also examined. We hypothesized that the later period after introduction of the initial NMT phase would be associated with lower 6-month motor severity.

## 2 Materials and methods

### 2.1 Study design

This retrospective, non-randomized historical cohort study used a sequential before-after comparison within a single clinical unit at a tertiary pediatric hospital. From June 2021 to January 2023, the unit followed a pathway consisting of diagnostic assessment and psychoeducation, followed by recommendations and referral for community-based physical and/or occupational therapy and psychological treatment when indicated. Patients treated during this period were classified as the Usual Care (UC) group. Beginning in February 2023, a specific in-hospital neuromotor treatment (NMT) was introduced as an additional component of the existing pathway. Thus, from February 2023 to June 2025, patients underwent diagnostic assessment and psychoeducation followed by a specialized hospital-based NMT program for FMD, after which recommendations and referral for community-based treatment were provided as in the previous pathway. The same clinical team and the same diagnostic criteria were applied consistently throughout both periods, without changes in the personnel responsible for diagnosis. Patients treated during this period were classified as the neuromotor-therapy-augmented (NMT-A) group. A more detailed description of the intervention is provided in Section 2.3.

The study was approved by the local institutional review board (CET Lazio Area 1; approval no. 8098/2025; 29 October 2025). The study used clinical data generated as part of routine care across the two sequential treatment periods. For patients whose clinical data had been collected before ethics approval, parents or legal guardians were contacted retrospectively and provided specific consent for the use of these previously collected clinical data for research purposes.

Clinical assessments were performed at diagnosis (T0) and at 6 months (T1) and 12 months (T2) thereafter. Data collection closed in June 2026. This study is reported in accordance with the STROBE guidelines for observational studies (see Supplementary Figure S1; Supplementary Table S1) (33).

### 2.2 Patients

Potentially eligible patients were identified through the clinical records of the participating unit across both study periods. Patients were included if they met three criteria: (1) FMD symptom onset before 19 years of age (observed range, 8–17 years); (2) a clinically definite diagnosis of FMD with motor disability according to Gupta and Lang criteria, with positive signs and distractibility maneuvers (6, 34), and one or more motor symptoms including weakness, tremor, dystonia, tics, jerks, or gait disorder; and (3) availability of Simplified Functional Movement Disorders Rating Scale (S-FMDRS) assessments at baseline (T0), 6 months (T1), and 12 months (T2). Records were excluded for possible/probable rather than clinically definite FMD, missing follow-up data, lack of consent for the use of clinical data for research purposes when such consent was required.

### 2.3 Sequential care periods and intervention

In both study groups, the same clinical unit provided positive diagnostic communication and psychoeducation regarding symptom mechanisms and prognosis, together with recommendations/referral for community-based physical and/or occupational therapy, psychotherapy when indicated, informational materials for families and community professionals, and counseling for local providers upon request. Pharmacological treatment was recommended according to clinical need, primarily for psychiatric comorbidities. Patients in the UC group proceeded directly from psychoeducation to community referral. Beginning in February 2023, a brief hospital-based neuromotor therapy (NMT) phase was added before the same community-referral framework, defining the NMT-A pathway.

Hospital-based NMT was delivered by a TNPEE with pediatric neurorehabilitation experience and specific training in pediatric movement disorders and FND. Treatment generally began within 15 days of diagnosis and comprised a median of 8 weekly 50-min sessions (range, 6–12). Established FMD-informed rehabilitation principles were organized across four domains: motor retraining through attentional modulation and implicit learning, proprioceptive and kinesthetic integration, regulation of bodily arousal, and postural control and balance. Content was developmentally adapted through play and age-appropriate tasks, school and social goals, consideration of neurodevelopmental vulnerabilities, and caregiver psychoeducation (14, 21, 22, 25–27, 31, 32). Further details on materials, home strategies, caregiver involvement, tailoring, progression and discharge, multidisciplinary review, and treatment fidelity are reported in Supplementary Table S2 using the Template for Intervention Description and Replication (TIDieR) framework (35).

After completion of the hospital-based NMT phase, recommendations and referral for community-based physical and/or occupational therapy and, when indicated, psychotherapy were provided according to the same community-referral framework used in the UC group. No random, referral-based, or study-defined severity criterion determined pathway assignment: the pathway available to each patient was fixed solely by the non-overlapping calendar period in which the diagnosis occurred. Follow-up assessments were conducted at 6 and 12 months. Community-treatment exposure was reconstructed retrospectively from family reports and checked against all clinical documentation available at follow-up. An intervention was classified as sustained when continued for at least 3 consecutive months, a pragmatic threshold defined before the present analyses to distinguish sustained from brief or isolated exposure and consistent with treatment durations described in FND rehabilitation literature (36, 37). Figure 1 summarizes the shared components, the structured addition introduced in the later period, the sequential calendar periods, and the assessment time points.

**Figure 1:**
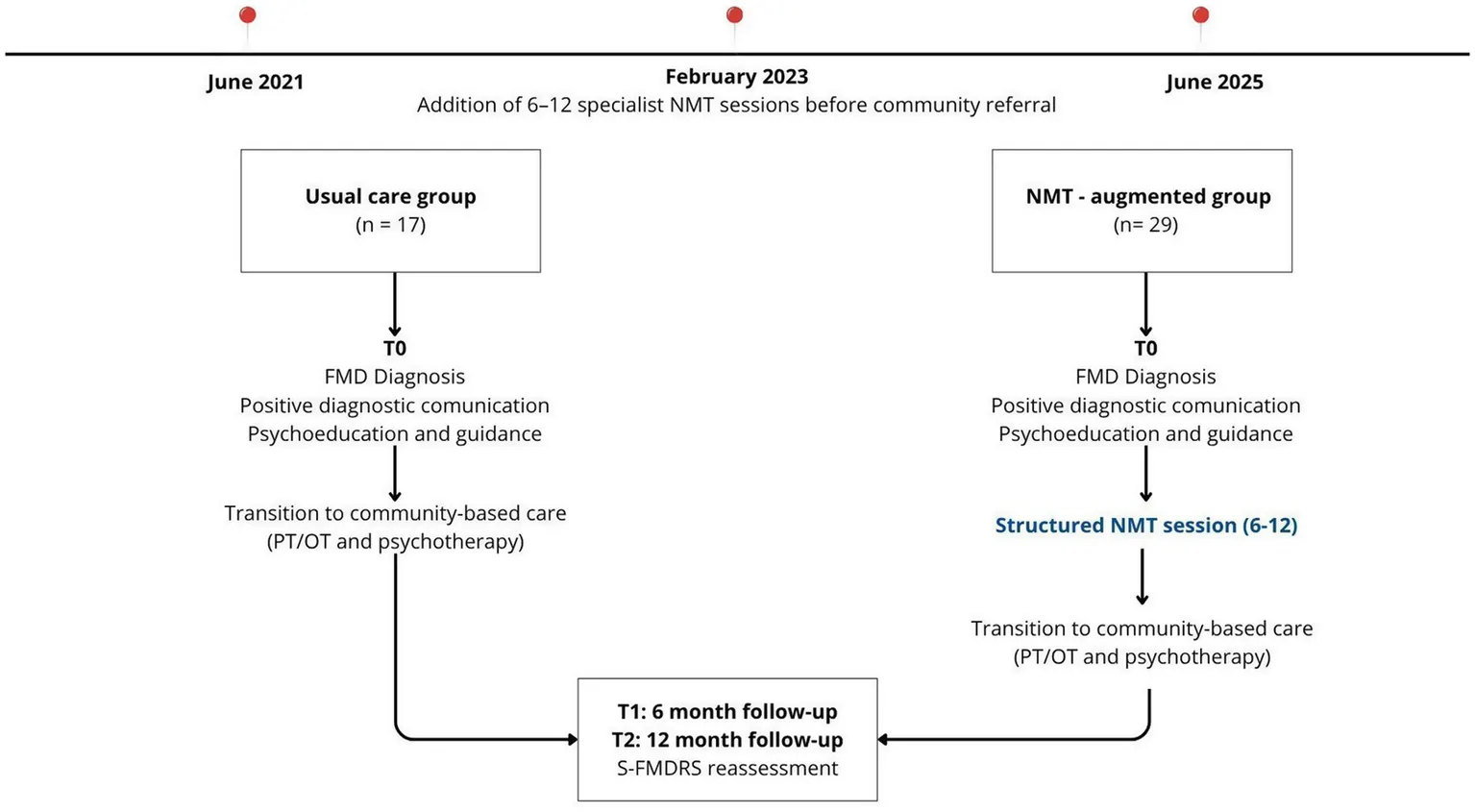
Study timeline showing sequential evolution of care within one clinical unit. During the UC period from June 2021 to January 2023, all patients received a positive FMD diagnosis, psychoeducation, and guidance for families, schools, and treating professionals, followed by recommendations/referral for community care. Beginning in February 2023, the same unit added 6–12 specialist NMT sessions before the same community-referral framework; this NMT-A period continued through June 2025. Suggested community treatment comprised physical and/or occupational therapy, with psychotherapy when indicated. S-FMDRS was reassessed at 6 months (T1) and 12 months (T2). FMD, Functional movement disorders; NMT, Neuromotor therapy; UC, Usual care; S-FMDRS, Simplified Functional Movement Disorders Rating Scale.

### 2.4 Outcomes

#### 2.4.1 Baseline demographic and clinical variables

Baseline demographic and clinical variables were extracted from medical records. These included age at FMD symptom onset, sex, the number of previous medical referrals before the definitive FMD diagnosis, symptom type and mode of onset, precipitating and predisposing factors, FMD phenotype, co-occurring functional neurological symptoms, comorbidities, and family history of neurological disease. Patients presenting with more than one motor phenotype were classified as having combined FMD. Based on the information collected regarding symptom type and mode of onset, disease duration was defined as the interval between the onset of the first FMD symptom and the definitive diagnosis, while symptom onset was classified as either acute - when symptoms appeared abruptly and clinical deterioration occurred over days or weeks - or slowly progressive (38). Documented precipitating factors included physical or psychological trauma, infection, adverse drug reactions, surgery, and general anesthesia. Predisposing adverse childhood experiences were recorded separately. Other functional neurological symptoms documented in the medical records included sensory symptoms, visual symptoms, and functional/dissociative seizures (FDS) (39). FDS were coded when explicitly documented in the medical record. Epilepsy was recorded separately; however, video-EEG confirmation and the level of diagnostic certainty were not systematically retained in the study dataset. Regarding comorbidities, both neurological and psychiatric conditions were recorded. Neurological comorbidities included migraine, epilepsy, neuropathy, hyperkinetic movement disorders, and other documented neurological conditions. Psychiatric comorbidities included anxiety disorders, major depressive disorder, somatic symptom disorders, neurodevelopmental disorders, eating disorders, schizophrenia-spectrum disorders, and impulse-control disorders. Other medical comorbidities and family history of neurological disease were recorded separately.

#### 2.4.2 Motor symptom severity and motor symptom-free status

Motor symptom severity was assessed at baseline, 6 months, and 12 months using the S-FMDRS (40). Assessments were performed by two neuropsychiatrists from the same clinical team, not blinded to treatment history, who had received the same initial training in standardized administration of the scale and remained involved across both study periods; neither delivered the hospital-based NMT intervention. Cases were discussed in multidisciplinary team meetings, which provided a shared clinical framework and supported consistency in scoring.

This clinician-rated instrument was derived from the Psychogenic Movement Disorders Rating Scale to provide a rapid and practical assessment of FMD severity. It rates the severity and duration of abnormal movements across seven body regions, together with gait and speech impairment, without requiring classification of movement phenomenology. Each severity and duration item is scored from 0 to 3, yielding a total score from 0 to 54; higher scores indicate greater motor symptom severity. The scale has demonstrated high inter-rater reliability, strong concurrent validity with the original rating scale, and sensitivity to clinical change (40). Although developed and validated in adults rather than specifically in pediatric populations, it has subsequently been applied in research cohorts that included participants aged 16–17 years (41, 42). The primary outcome was the continuous S-FMDRS score at 6 months. Motor symptom-free status, defined as an S-FMDRS score of 0, was evaluated as a secondary exploratory outcome. Both outcomes were reassessed at 12 months.

### 2.5 Statistical analysis

Baseline characteristics were summarized using mean (SD), median (IQR), or n (%), as appropriate; absolute standardized differences (ASDs) were reported alongside baseline *p* values to describe the magnitude of between-period imbalance. The primary analysis compared 6-month S-FMDRS scores using linear regression adjusted for baseline S-FMDRS and disease duration, with HC3 heteroscedasticity-consistent standard errors. Baseline S-FMDRS was selected because it is the baseline value of the outcome, and disease duration because it is a clinically relevant pre-pathway prognostic factor in pediatric FND/FMD (43, 44); alternative specification of disease duration was examined in sensitivity analyses. Functional weakness was examined as an additional sensitivity covariate because it showed a clinically meaningful baseline imbalance and could plausibly influence motor outcome; the primary adjustment set was kept parsimonious given the limited sample size. Because pathway and calendar period were perfectly collinear, all pathway coefficients also represent calendar-period contrasts. Exploratory S-FMDRS-defined motor symptom-free status at 6 and 12 months was summarized using observed risks and Newcombe risk differences; Firth penalized logistic regression (45) was used at 6 months because of sparse events, whereas an ordinary logistic model with average marginal effects was used at 12 months. A supportive linear mixed-effects model examined post-baseline S-FMDRS scores at 6 and 12 months. Analyses were complete-case, with no outcome imputation. Additional analyses addressing covariate specification, the marked lower-bound accumulation of S-FMDRS values, baseline imbalance, and potential attrition bias are detailed in Supplementary Tables S3–S5; Supplementary Figure S2. Community-care engagement outcomes were compared using two-sided Fisher’s exact tests with absolute and relative effect estimates. No *a priori* sample-size calculation was performed, and no multiplicity adjustment was applied to exploratory or supportive analyses. Analyses were performed using Jamovi version 2.7 (46), with two-sided significance set at *p* < 0.05.

## 3 Results

### 3.1 Study flow and baseline characteristics

Of the 63 records screened at a single clinical unit, 25 were from the UC period and 38 from the NMT-A period. In the UC period, 8 records were excluded: 6 because follow-up data were unavailable and 2 because diagnostic certainty was classified as possible or probable. In the NMT-A period, 9 records were excluded: 4 because follow-up data were unavailable, 3 because diagnostic certainty was classified as possible or probable, and 2 because consent for the research use of previously collected clinical data was not provided. The final analytical sample with complete 6- and 12-month S-FMDRS follow-up therefore comprised 46 patients: 17 of 25 screened during the UC period and 29 of 38 screened during the NMT-A period.

Baseline characteristics are summarized in Table 1. Disease duration was shorter in the NMT-A group than in the UC group [median (IQR), 0.2 (0.4) vs. 0.9 (1.7) years; *p* = 0.007], and visual disorders were documented in 7 of 29 NMT-A patients (24.1%) and none of the 17 UC patients (*p* = 0.036). Absolute standardized differences additionally indicated between-period imbalance in functional weakness, tremor, baseline S-FMDRS, age at diagnostic assessment, and gait disorders, irrespective of statistical significance. Among the 10 patients excluded because follow-up data were unavailable, baseline age, sex, and S-FMDRS did not differ statistically from those of analyzed participants, although baseline S-FMDRS was descriptively more variable (Supplementary Table S5).

**Table 1:** Baseline demographic and clinical characteristics of children and adolescents with FMD in the NMT-A and UC groups, treated sequentially within one clinical unit.

Variables	Total FMD sample *n* = 46	NMT-A group *n* = 29	UC group *n* = 17	*p* value NMT-A vs. UC	Absolute ASD
Female sex, *n* (%)	29 (63.0)	17 (58.6)	12 (70.6)	0.533^F^	0.25
Age at diagnostic assessment, mean (SD)	14.2 (2.3)	13.8 (2.3)	14.9 (2.1)	0.126^T^	0.48
Age at FMD symptom onset, mean (SD)	13.3 (2.0)	13.1 (1.9)	13.6 (2.2)	0.435^T^	0.24
Number of previous referrals, median (IQR)	1.0 (1.75)	1.0 (1.0)	1.0 (1.0)	0.267^M^	0.23
Disease duration (years), median (IQR)	0.3 (0.9)	0.2 (0.4)	0.9 (1.7)	**0.007** ^**M**^	0.46
Acute onset of symptoms, *n* (%)	31 (67.4)	21 (72.4)	10 (58.8)	0.516^F^	0.29
Definite precipitating factor, *n* (%)	36 (78.3)	22 (75.9)	14 (82.4)	0.723^F^	0.16
FMD phenotype, *n* (%)					
Combined FMD	28 (60.9)	19 (65.5)	9 (52.9)	0.533^F^	0.26
Weakness	28 (60.9)	21 (72.4)	7 (41.2)	0.060^F^	0.66
Dystonia	3 (6.5)	1 (3.4)	2 (11.8)	0.545^F^	0.32
Jerks	8 (17.4)	4 (13.8)	4 (23.5)	0.443^F^	0.25
Tremor	11 (23.9)	4 (13.8)	7 (41.2)	0.070^F^	0.64
Tics	4 (8.7)	2 (6.9)	2 (11.8)	0.619^F^	0.17
Gait disorders	33 (71.7)	23 (79.3)	10 (58.8)	0.181^F^	0.45
Other FND symptoms, *n* (%)					
Sensory symptoms	20 (43.5)	11 (37.9)	9 (52.9)	0.369^F^	0.30
Visual disorders	7 (15.2)	7 (24.1)	0 (0.0)	**0.036** ^**F**^	0.80
Functional/dissociative seizures	10 (21.7)	7 (24.1)	3 (17.6)	0.723^F^	0.16
Neurological comorbidities, *n* (%)	8 (17.4)	5 (17.2)	3 (17.6)	1.000^F^	0.01
Migraine	2 (4.3)	2 (6.9)	0 (0.0)	0.524^F^	0.38
Epilepsy	2 (4.3)	1 (3.4)	1 (5.9)	1.000^F^	0.12
Other neurological comorbidities §	4 (8.7)	2 (6.9)	2 (11.8)	0.619^F^	0.17
Psychiatric comorbidities, *n* (%)	22 (47.8)	13 (44.8)	9 (52.9)	0.761^F^	0.16
Other medical comorbidities, *n* (%)	19 (41.3)	12 (41.4)	7 (41.2)	1.000^F^	0.00
Family history of neurological disease, *n* (%)	11 (23.9)	7 (24.1)	4 (23.5)	1.000^F^	0.01
Predisposing ACEs, *n* (%)	15 (32.6)	8 (27.6)	7 (41.2)	0.516^F^	0.29
Baseline S-FMDRS, median (IQR)	10.0 (4.0)	10.0 (4.0)	8.0 (2.0)	0.107^M^	0.56

§ Other neurological comorbidities included hyperkinetic movement disorder (*n* = 1), neuropathy (*n* = 1), and other unspecified neurological diagnoses (*n* = 2). ACEs, adverse childhood experiences; F, two-sided Fisher’s exact test; ASD, absolute standardized difference (values were calculated on the original continuous scale for continuous variables and from group proportions for binary variables); FMD, functional movement disorder; IQR, interquartile range; M, Mann–Whitney U test; *n*, number; NMT-A, neuromotor-therapy-augmented pathway; UC, usual care; S-FMDRS, Simplified Functional Movement Disorders Rating Scale; SD, standard deviation; T, independent-samples *t* test. Statistically significant values (*p* < 0.05) are reported in bold.

### 3.2 Treatment delivery and community-care engagement

The NMT protocol comprised a median of eight weekly 50-min sessions (range, 6–12). No adverse events or complications leading to early discontinuation were documented in the available clinical records; adverse events were not collected through a structured prospective surveillance protocol.

At the 12-month assessment, sustained community-based physical and/or occupational therapy was reported in 21 of 28 patients with available data in the NMT-A group (75.0%), compared with 3 of 17 patients in the UC group (17.6%; *p* < 0.001). Sustained psychotherapy was reported in 18 of 28 patients in the NMT-A group (64.3%) and 14 of 17 in the UC group (82.4%, *p* = 0.311). Pharmacological treatment was recorded in 10 of 29 patients in the NMT-A group (34.5%) versus 12 of 17 in the UC group (70.6%, *p* = 0.031). Full effect estimates are reported in Table 2.

**Table 2:** Community-care engagement and post-diagnostic treatment exposure documented for at least 3 consecutive months by the 12-month assessment (T2) in the sequential calendar-period cohorts.

Variable	NMT-A group n/N (%)	UC group n/N (%)	RD, percentage points (95% CI)	RR (95% CI)	*p*
Community-care engagement					
Physical and occupational therapy	21/28 (75.0)	3/17 (17.6)	57.4 (27.6 to 74.2)	4.25 (1.74 to 12.46)	**<0.001**
Psychotherapy	18/28 (64.3)	14/17 (82.4)	−18.1 (−39.8 to 9.7)	0.78 (0.53 to 1.17)	0.311
Post-diagnostic treatment exposure					
Pharmacological treatment	10/29 (34.5)	12/17 (70.6)	−36.1 (−57.8 to −6.2)	0.49 (0.26 to 0.89)	**0.031**

Values are n/N (%). Risk differences are expressed in percentage points and calculated as NMT-A minus UC; risk ratios compare the NMT-A period with the UC period. *p* values are from two-sided Fisher’s exact tests. One NMT-A participant had missing physical and/or occupational therapy and psychotherapy data. CI, confidence interval; NMT-A, neuromotor-therapy-augmented pathway; UC, usual care; RD, risk difference; RR, risk ratio. Statistically significant values (*p* < 0.05) are reported in bold.

### 3.3 Motor outcomes at 6 months

#### 3.3.1 Motor symptom severity

At 6 months, the observed mean S-FMDRS score was 3.86 (SD 5.23) in the NMT-A group and 4.76 (SD 2.91) in the UC group. In the primary robust regression adjusted for baseline S-FMDRS and disease duration, the estimated mean score was 3.58 (95% CI 1.84–5.32) in NMT-A and 5.24 (95% CI 3.59–6.90) in UC. The adjusted NMT-A-minus-UC mean difference was −1.66 points (95% CI -4.04 to 0.72; *p* = 0.166). Higher baseline S-FMDRS was associated with greater 6-month severity (B = 0.69 per baseline point; 95% CI 0.43–0.95; *p* < 0.001). Sensitivity analyses addressing covariate specification and outcome distribution yielded the same overall interpretation (Supplementary Table S4); raw individual S-FMDRS trajectories are shown in Supplementary Figure S2.

#### 3.3.2 Motor symptom-free status

At 6 months, S-FMDRS-defined motor symptom-free status was observed in 16 of 29 patients in the NMT-A group (55.2%) and 1 of 17 patients in the UC group (5.9%). The unadjusted absolute risk difference (NMT-A minus UC) was 49.3 percentage points (95% Newcombe CI 21.8 to 66.4). In the Firth model adjusted for baseline S-FMDRS and disease duration, the adjusted OR was 26.49 (95% profile-likelihood CI 4.28–326.99; penalized likelihood-ratio *p* < 0.001). Because the UC estimate was based on a single event, the adjusted OR was imprecise and is presented secondary to the absolute risk difference; crude and sensitivity estimates are reported in Supplementary Table S4.

### 3.4 Motor outcomes at 12 months and longitudinal course

At 12 months, S-FMDRS-defined motor symptom-free status was observed in 25 of 29 patients in the NMT-A group (86.2%) and 9 of 17 patients in the UC group (52.9%). The unadjusted absolute risk difference was 33.3 percentage points (95% Newcombe CI 6.5 to 56.8). In the exploratory logistic model adjusted for baseline S-FMDRS and disease duration, the average marginal effect corresponded to an adjusted risk difference of 42.1 percentage points (95% CI 16.8 to 67.5; *p* = 0.001); the corresponding adjusted OR is reported in Supplementary Table S4. In the supportive mixed-effects model, S-FMDRS decreased further from 6 to 12 months (*p* < 0.001), whereas neither the overall pathway-period effect (*p* = 0.204) nor the pathway-period-by-time interaction (*p* = 0.526) was significant. In extreme-case analyses for the 10 unavailable follow-up outcomes, risk differences remained directionally positive in the least-favorable scenario for NMT-A, but the confidence intervals included zero (Supplementary Table S5). Observed motor symptom-free proportions at both follow-up points are shown in Figure 2.

**Figure 2:**
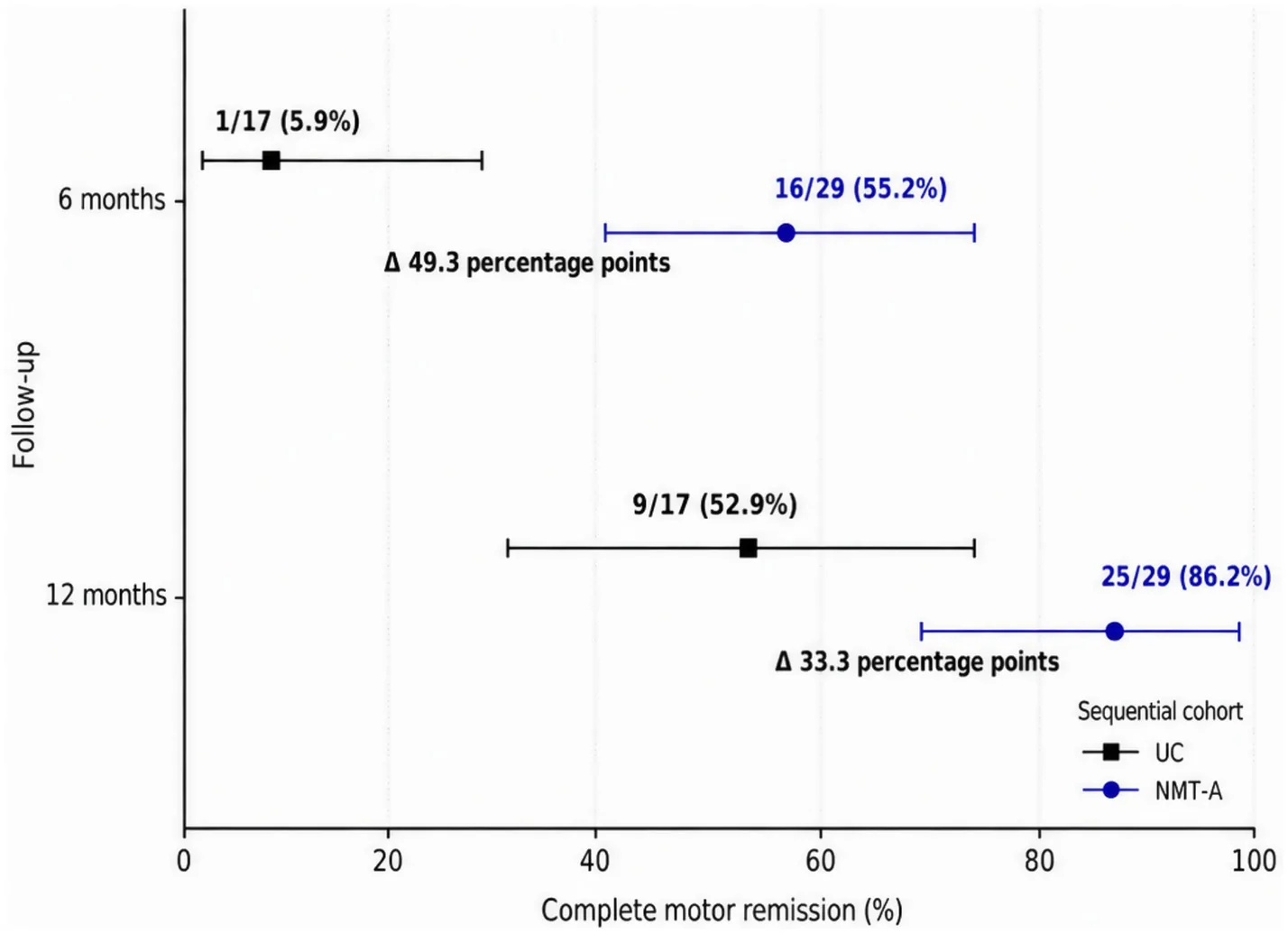
S-FMDRS-defined motor symptom-free status in the UC and NMT-A groups at 6- and 12-month follow-up. The cohorts were treated sequentially, not concurrently. Motor symptom-free status was defined as an S-FMDRS total score of 0 at the follow-up examination and therefore represents absence of observable motor manifestations at that assessment rather than sustained global recovery. Points indicate observed proportions; error bars indicate 95% Wilson confidence intervals. At 6 months, motor symptom-free status was observed in 16/29 NMT-A patients (55.2%) and 1/17 UC patients (5.9%). At 12 months, it was observed in 25/29 NMT-A patients (86.2%) and 9/17 UC patients (52.9%).

## 4 Discussion

### 4.1 Principal findings and interpretation

The primary hypothesis was not confirmed. At 6 months, the adjusted mean S-FMDRS score was 1.66 points lower in the NMT-A period than in UC, but the 95% confidence interval (−4.04 to 0.72) included the null. This interval is compatible with anything from a potentially relevant reduction in mean motor severity to little or no between-period difference; accordingly, the nonsignificant result should not be interpreted as evidence of equivalence or absence of benefit. Interpretation is further limited by the absence of an *a priori* sample-size calculation and by the lack of a pediatric minimal clinically important difference for the S-FMDRS.

The secondary exploratory endpoint provided a different view of the 6-month outcome: motor symptom-free status was observed in 55.2% of NMT-A participants and 5.9% of UC participants (risk difference, +49.3 percentage points). This finding is clinically notable but should remain hypothesis-generating because the endpoint was exploratory and was not multiplicity-adjusted. The apparent divergence from the continuous outcome is plausibly related to the floor effect and to heterogeneity in residual severity. An S-FMDRS score of 0 is the natural lower bound of the scale and indicates no observable motor manifestations at that assessment, but it is a stringent dichotomization that has not been validated as a pediatric recovery threshold; even a score of 1 or 2 is classified as not motor symptom-free. Thus, a large difference in the proportion reaching the scale floor can coexist with a much smaller difference in mean severity across the full cohort.

At 12 months, motor symptom-free status remained more frequent in NMT-A than in UC (86.2% vs. 52.9%), while mean S-FMDRS scores were low in both groups. The absolute risk difference narrowed from 49.3 percentage points at 6 months to 33.3 points at 12 months. The nonsignificant pathway-by-time interaction should not be interpreted as evidence that trajectories were equivalent; with 46 analyzed participants, the study simply did not demonstrate differential post-baseline change. The increase in motor symptom-free status in UC from 5.9% at 6 months to 52.9% at 12 months is itself clinically important and suggests substantial later improvement in the earlier cohort.

### 4.2 Findings in context and natural history

Substantial improvement in both periods is consistent with the generally favorable course reported in pediatric FND/FMD cohorts and rehabilitation programs (14–16, 19, 22). Published pediatric studies vary considerably in setting, treatment intensity, follow-up duration, and definitions of recovery, but many describe marked improvement or symptom resolution over months. Against this background, the later convergence of the UC cohort is compatible with the natural history of pediatric FMD and/or benefit from care received after diagnosis, whereas the earlier separation between periods raises the question of whether the organization and timing of the later pathway influenced when some patients reached the scale floor. Because outcomes were assessed only at 6 and 12 months, the present data cannot distinguish these explanations or establish actual time to recovery.

The baseline S-FMDRS values were broadly within ranges reported in predominantly adult FMD cohorts (41, 47, 48), but direct age-specific comparison remains uncertain because the scale was developed and validated in adults and has not been specifically validated in pediatric populations. Disease duration also deserves particular attention. It was shorter in the NMT-A period, and recent pediatric studies have associated longer symptom duration or delayed FMD-directed treatment with greater subsequent motor severity (43, 44). These findings support the clinical relevance of timely assessment and treatment, but in the present historical comparison the same imbalance may also mark a change in referral timing or case-mix and therefore remains a source of residual confounding rather than evidence for an NMT-specific mechanism.

### 4.3 Possible pathway mechanisms

Any mechanistic interpretation is necessarily provisional because no mediator was measured. One plausible pathway-level explanation is that introducing FMD-informed motor practice soon after diagnosis may help translate diagnostic understanding into functional experience before transition to community care. The NMT phase used attentional modulation and implicit motor learning, sensorimotor and proprioceptive strategies, regulation of bodily arousal, and graded postural and functional practice. These components are consistent with contemporary models of altered attention, motor prediction, sensory integration, and agency in FMD and with rehabilitation principles aimed at restoring automatic movement (1, 4–10, 26, 27). Their developmental adaptation may also be relevant in children and adolescents, but the present study did not test these mechanisms or compare professional models of rehabilitation.

The greater documented engagement with community motor rehabilitation in the later period may represent another pathway-level difference worth prospective study. A brief specialist phase could conceivably facilitate handover by reinforcing diagnostic acceptance, establishing an initial motor-retraining framework, and giving families and community clinicians a clearer rehabilitation plan. However, the present data cannot establish this sequence: treatment initiation and discontinuation dates were not reliably available, and sustained engagement was reconstructed cumulatively by the 12-month assessment. Community treatment may therefore have preceded, followed, or changed in response to motor improvement, making reverse causation possible.

### 4.4 Limitations of the study

The main limitation is the non-concurrent historical design. Pathway and calendar period were perfectly collinear, so an NMT-specific causal effect cannot be separated from secular change. The shorter disease duration in NMT-A may reflect earlier recognition and referral, but it may also indicate a period-related change in case-mix; covariate adjustment reduces but cannot eliminate this problem. The UC period also overlapped more extensively with the COVID-19 pandemic and its aftermath, when community-service availability may have differed from the later period. Post-diagnostic treatment exposures differed as well: community motor rehabilitation was much more frequent in NMT-A, whereas pharmacological treatment was more frequent in UC and psychotherapy exposure also differed descriptively. These variables may reflect treatment need, referral practice, service availability, or downstream effects of recovery and cannot be cleanly treated as baseline confounders or mediators without reliable temporal information.

Selection into the analytical sample is another concern. Follow-up data were unavailable for 6 of 25 screened UC records (24.0%) and 4 of 38 NMT-A records (10.5%), and eligibility for the primary analysis required available S-FMDRS assessments at both 6 and 12 months. This may preferentially retain families with greater follow-up engagement, access to care, or particular clinical trajectories. Baseline comparisons of analyzed and excluded participants were necessarily underpowered, and the extreme-case sensitivity analyses were informative but did not eliminate this uncertainty: under the least-favorable assumptions, risk differences remained directionally positive but their confidence intervals included zero.

Statistical precision was limited by the modest and unequal cohort sizes. No *a priori* sample-size calculation was performed, and no dated prespecified statistical analysis plan was available. The continuous 6-month S-FMDRS was therefore treated as the primary outcome in the present analytic framework, while binary motor symptom-free ana stations at one assessment rather than sustained or global recovery. These considerations favor emphasis on effect estimates, confidence intervals, raw score distributions, and convergence across sensitivity analyses rather than on isolated *p* values.

Measurement and ascertainment limitations also remain. The same two trained neuropsychiatrists assessed both periods, which provided procedural continuity, but they were not blinded to treatment history; because they were members of the treating team, expectancy or detection bias cannot be excluded. No formal inter-rater reliability checks or blinded reassessment were performed, and rater drift over the multi-year study period is possible. Community-treatment exposure relied on family report cross-checked against available documentation and remains vulnerable to recall or misclassification. Broader outcomes such as participation, school functioning, quality of life, psychological symptoms, and family functioning were not systematically measured. Finally, adverse events were not collected through structured prospective surveillance, so the absence of documented events cannot establish safety.

### 4.5 Strengths of the study

Strengths include comparison of two clearly defined care configurations within the same clinical unit, standardized S-FMDRS assessments at fixed 6- and 12-month time points, explicit characterization of baseline imbalance and attrition, and transparent sensitivity analyses addressing sparse events, model specification, the floor effect, and missing follow-up. Raw individual trajectories are provided in the Supplementary material, and the specialist intervention and its developmental adaptations are described in sufficient operational detail, including through the TIDieR-based Supplementary Table S2, to support prospective evaluation.

### 4.6 Clinical implications

The findings do not establish the efficacy of NMT as an isolated intervention, nor do they demonstrate cost-effectiveness or scalability. They do, however, show that a brief specialist neuromotor phase can be embedded within routine pediatric FMD care before transition to community services. The large exploratory between-period difference in 6-month motor symptom-free status and the greater continuity of community motor rehabilitation make this configuration clinically relevant for further study, particularly in services seeking to bridge the gap between specialist diagnosis and local rehabilitation. The developmental focus of the pathway - including play-based practice, attention to neurodevelopmental vulnerabilities, school and social goals, and caregiver psychoeducation - provides one model for adapting established FMD rehabilitation principles to pediatric care.

### 4.7 Future research directions

Prospective studies should use contemporaneous comparison groups and a dated analysis plan, with sample-size calculations based on a clinically interpretable continuous motor outcome. Repeated assessments or time-to-event methods are needed to determine actual time to motor recovery rather than status at two scheduled visits. Future work should also validate pediatric outcome measures and clinically meaningful thresholds, prospectively record the timing, dose, fidelity, and discontinuation of community rehabilitation and co-interventions, and distinguish treatment exposure that precedes outcome from treatment that changes in response to recovery. Broader endpoints should include participation, school functioning, quality of life, patient- and caregiver-reported recovery, psychological symptoms, health-care utilization, acceptability, and costs. Such designs would allow testing of whether an early specialist phase independently influences motor recovery, transition to community care, or both (49, 50).

## 5 Conclusion

The primary hypothesis of lower adjusted mean S-FMDRS severity at 6 months in the later NMT-A period was not confirmed, although the confidence intAccess to specialized, FMD-informed rehabilitation remains limited for children with functional movement disorders, and comparative data on brief, low-intensity models are scarce. In this single-center historical cohort, the primary hypothesis of lower adjusted mean S-FMDRS at 6 months in the NMT-A period was not confirmed, although the confidence interval did not exclude a potentially relevant reduction. In exploratory analyses, this treatment period was associated with a higher proportion of patients who were motor symptom-free at 6 months and with greater continuity of community motor rehabilitation, while both cohorts improved considerably by 12 months, consistent with a favorable natural course. Because pathway and calendar period were inseparable, however, these findings reflect the overall later care pathway rather than an isolated effect of NMT. They nevertheless provide preliminary data supporting prospective, contemporaneously controlled studies to test whether a brief specialist-to-community pathway improves the probability or timing of motor recovery and the transition to community rehabilitation in pediatric FMD.s but are available from the corresponding author upon reasonable request, subject to institutional and ethics requirements.

## Data Availability

The raw data supporting the conclusions of this article will be made available by the authors, without undue reservation.
