# Supplementary material for: A brief neuromotor-therapy-augmented pathway for pediatric functional movement disorders: a sequential historical cohort study
Source: Front Neurol. 2026 Oct 30;17:1968237. doi: 10.3389/fneur.2026.1968237 (PMC13759379; doi:10.3389/fneur.2026.1968237)
Supplement: Supplementary file 1 [file Supplementary_file_1.docx]

**Supplementary Material**

**Supplementary Methods (STROBE)**

**Supplementary Figure 1S. STROBE flowchart for participant selection.**

**Supplementary Table 1S. STROBE Checklist.**

| **Item** | **Item Description** | **Location (or reason for not reporting)** |
| --- | --- | --- |
| **Title and abstract** | | |
| 1a. Indicate the study’s design | Indicate the study’s design with a commonly used term in the title or the abstract. | Title and Abstract. |
| 1b. Abstract | Provide in the abstract an informative and balanced summary of what was done and what was found. | Abstract. |
| **Introduction** | | |
| 2. Background / rationale | Explain the scientific background and rationale for the investigation being reported. | Introduction. |
| 3. Objectives | State specific objectives, including any prespecified hypotheses. | Introduction. |
| **Methods** | | |
| 4. Study design | Present key elements of study design early in the paper. | Methods 2.1. |
| 5. Setting | Describe the setting, locations, and relevant dates, including periods of recruitment, exposure, follow-up, and data collection. | Methods 2.1-2.3. |
| 6a. Eligibility criteria | Cohort study: Give the eligibility criteria, and the sources and methods of selection of participants. Describe methods of follow-up. Case-control study: Give the eligibility criteria, and the sources and methods of case ascertainment and control selection. Give the rationale for the choice of cases and controls. Cross-sectional study: Give the eligibility criteria, and the sources and methods of selection of participants. | Methods 2.1-2.2. |
| 6b. Matching criteria | Cohort study: For matched studies, give matching criteria and number of exposed and unexposed. Case-control study: For matched studies, give matching criteria and the number of controls per case. | Not applicable: the period-defined cohorts were not matched. |
| 7. Variables | Clearly define all outcomes, exposures, predictors, potential confounders, and effect modifiers. Give diagnostic criteria, if applicable. | Methods 2.1 and 2.3-2.5. |
| 8. Data sources / measurement | For each variable of interest give sources of data and details of methods of assessment (measurement). Describe comparability of assessment methods if there is more than one group. | Partial: Methods 2.1 and 2.3-2.4; Discussion 4.4. |
| 9. Bias | Describe any efforts to address potential sources of bias. | Methods 2.1, 2.3 and 2.5; Discussion 4.4; Supplementary Table 5S. |
| 10. Study size | Explain how the study size was arrived at. | Methods 2.5: all eligible records meeting the complete 6- and 12-month S-FMDRS availability criterion were included; no a priori calculation. |
| 11. Quantitative variables | Explain how quantitative variables were handled in the analyses. If applicable, describe which groupings were chosen, and why. | Methods 2.3-2.5. |
| 12a. Statistical methods | Describe all statistical methods, including those used to control for confounding. | Methods 2.5. |
| 12b. Statistical methods – subgroups and interactions | Describe any methods used to examine subgroups and interactions. | Methods 2.5 (pathway-by-time interaction and additional-adjustment analysis). |
| 12c. Statistical methods – missing data | Explain how missing data were addressed. | Methods 2.2, and 2.5; Results 3.1; Table 2. Complete-case selection, available-case handling, absence of imputation, follow-up exclusions, and the single item-level missing record are reported. |
| 12di. Statistical methods – loss to follow-up | Cohort study: If applicable, describe how loss to follow-up was addressed. | Methods 2.2, and 2.5; Results 3.1; Discussion 4.4; Supplementary Table 5S. Records with unavailable 6- or 12-month S-FMDRS follow-up were excluded; period-specific counts and attrition sensitivity analyses are reported. |
| 12dii. Statistical methods – matching cases and controls | Case-control study: If applicable, explain how matching of cases and controls was addressed. | Not applicable: this was not a matched case-control study. |
| 12diii. Statistical methods – sampling strategy | Cross-sectional study: If applicable, describe analytical methods taking account of sampling strategy. | Not applicable: this was not a cross-sectional survey with a sampling design. |
| 12e. Statistical methods – sensitivity analyses | Describe any sensitivity analyses. | Methods 2.5; Results 3.3-3.4; Discussion 4.4-4.5, Supplementary Tables 4S-5S. |
| **Results** | | |
| 13a. Participant numbers | Report the numbers of individuals at each stage of the study—e.g., numbers potentially eligible, examined for eligibility, confirmed eligible, included in the study, completing follow-up, and analysed; Consider use of a flow diagram. | Results 3.1; Supplementary Figure 1S (63 assessed, 17 excluded, 46 included and analysed: 17 UC and 29 NMT-A). |
| 13b. Participants – non-participation | Give reasons for non-participation at each stage. | Results 3.1; Supplementary Figure 1S (reasons for all 17 exclusions are reported). |
| 13c. Participants – flow diagram | Consider use of a flow diagram. | Supplementary Figure 1S (participant flow diagram). |
| 14a. Descriptive data – participant characteristics | Give characteristics of study participants (e.g., demographic, clinical, social) and information on exposures and potential confounders. Present the information in a table. | Results 3.1; Table 1. |
| 14b. Descriptive data – missing data | Indicate the number of participants with missing data for each variable of interest. | Table 2, identifies the single missing NMT-A record for both community-engagement variables. |
| 14c. Descriptive data – follow-up time | Cohort study: Summarise follow-up time—e.g., average and total amount. | Methods 2.1, 2.3 and 2.4.2, fixed assessments at 6 and 12 months. |
| 15. Outcome data | Cohort study: Report numbers of outcome events or summary measures over time. Case-control study: Report numbers in each exposure category, or summary measures of exposure. Cross-sectional study: Report numbers of outcome events or summary measures. | Results 3.3-3.4; Figure 2, p. 17. Six- and 12-month motor symptom-free counts and continuous S-FMDRS summaries are reported. |
| 16a. Main results | Give unadjusted estimates and, if applicable, confounder-adjusted estimates and their precision (e.g., 95% confidence intervals). Make clear which confounders were adjusted for and why they were included. | Abstract, p. 1; Methods 2.5; Results 3.2-3.4; Table 2. Unadjusted and adjusted estimates, 95% CIs, and adjustment covariates are reported. |
| 16b. Main results – category boundaries | Report category boundaries when continuous variables were categorised. | Methods 2.3-2.4: sustained care was defined as at least 3 months; motor symptom-free status as S-FMDRS = 0; acute onset was operationally defined. |
| 16c. Main results – risk | If relevant, consider translating estimates of relative risk into absolute risk for a meaningful time period. | Abstract, p. 1; Results 3.3-3.4; Table 2. Absolute risk differences with 95% CIs are reported for motor symptom-free status at 6 and 12 months and community-care outcomes. |
| 17. Other analyses | Report other analyses done—e.g., analyses of subgroups and interactions, and sensitivity analyses. | Results 3.3-3.4; Supplementary Tables 4S-5S. Six-month sensitivity analyses, exploratory 12-month logistic regression, longitudinal mixed-effects analyses, and attrition sensitivity analyses are reported. |
| **Discussion** | | |
| 18. Key results | Summarise key results with reference to study objectives. | Discussion 4.1. |
| 19. Limitations | Discuss limitations of the study, taking into account sources of potential bias or imprecision. Discuss both direction and magnitude of any potential bias. | Discussion 4.4. |
| 20. Interpretation | Give a cautious overall interpretation considering objectives, limitations, multiplicity of analyses, results from similar studies, and other relevant evidence. | Discussion 4.1-4.7. |
| 21. Generalisability | Discuss the generalisability (external validity) of the study results. | Partial: Discussion 4.2 and 4., provide clinical and implementation context; external generalisability beyond this single tertiary Italian unit is not explicitly assessed. |
| **Other information** | | |
| 22. Funding | Give the source of funding and the role of the funders for the present study and, if applicable, for the original study on which the present article is based. | Funding: no financial support was received; funder role therefore not applicable. |

**Supplementary Intervention Description**

**Supplementary Table 2S.** TIDieR-style description of the hospital-based neuromotor therapy phase.

| TIDieR item | Description |
| --- | --- |
| 1. Brief name | Brief, developmentally adapted neuromotor therapy (NMT) phase delivered within the NMT-A care pathway. |
| 2. Why | To initiate FMD-informed motor relearning, promote automatic and functional movement, and prepare transition to community-based rehabilitation. |
| 3. Materials | Informational materials were available for families and community professionals. Between-session practice consisted of individualized verbal strategies rather than a standardized written home-exercise program. |
| 4. Procedures | Treatment followed four core domains: motor retraining through attentional modulation and implicit learning; proprioceptive and kinesthetic integration; regulation of bodily arousal; and reconstruction of postural control and balance. Treatment ended after recovery and stabilization of the target function, or after acquisition of the proposed strategies with readiness for community transition. |
| 5. Who provided | NMT was delivered by a Neuro and Psychomotor Therapist of Developmental Age (TNPEE) within the multidisciplinary pediatric team. The treating clinician had more than 20 years of continuous experience in pediatric neurorehabilitation and specific training in pediatric movement disorders, including pediatric FND. |
| 6. How | Individual face-to-face sessions were delivered within the specialist hospital service. Parents did not routinely attend sessions; they received post-session feedback and dedicated psychoeducation before and at the end of the NMT phase. Cases were periodically reviewed in multidisciplinary team meetings. |
| 7. Where | Multidisciplinary clinical unit at a tertiary pediatric hospital. |
| 8. When and how much | NMT generally began within 15 days of diagnosis while the biopsychosocial formulation was being refined. Sessions were approximately weekly and 50 minutes long; the median number was 8 (range, 6–12). Session number was individualized according to clinical response, functional goal attainment, symptom complexity/severity, and organizational feasibility for the service and family. |
| 9. Tailoring | The four-domain framework was individualized to symptoms and developmental profile. Adaptations included extensive use of play and age-appropriate pediatric neurorehabilitation strategies, school and social goals, consideration of co-occurring neurodevelopmental vulnerabilities, and caregiver psychoeducation. |
| 10. Modifications | No study-wide protocol modification was introduced during the NMT-A period. Within-patient content was adapted over time according to symptoms, functional response, and multidisciplinary case review. |
| 11. How well – planned | The four therapeutic domains provided a common framework, with individualized treatment planning and periodic multidisciplinary review. No formal treatment-fidelity checklist was used. |
| 12. How well – actual | NMT was delivered as routine clinical care. The number of sessions was recorded, but participant-level adherence to specific intervention components or a formal fidelity score was not collected. |

**Supplementary Statistical Methods**

**Supplementary Table 3S**. Statistical analysis framework.

| **Analysis** | **Outcome** | **Model/test** | **Adjustment/estimator** | **Role** |
| --- | --- | --- | --- | --- |
| Primary 6-month continuous | S-FMDRS at 6 months | Linear regression | Baseline S-FMDRS + disease duration; HC3 SEs | Primary |
| Covariate sensitivity | S-FMDRS at 6 months | Linear regression | Primary covariates + functional weakness; HC3 | Sensitivity |
| Functional-form sensitivity | S-FMDRS at 6 months | Linear regression | Baseline S-FMDRS + log-transformed disease duration; HC3 | Sensitivity |
| Distributional sensitivity | S-FMDRS at 6 months | Non-parametric bootstrap | Same adjusted model; 10,000 BCa resamples | Sensitivity |
| Non-parametric sensitivity | S-FMDRS at 6 months | Mann-Whitney U | Unadjusted; rank-biserial correlation | Sensitivity |
| 6-month motor symptom-free status | S-FMDRS = 0 at 6 months | Fisher/Newcombe + Firth logistic | Firth adjusted for baseline S-FMDRS + disease duration; weakness sensitivity | Exploratory |
| 12-month motor symptom-free status | S-FMDRS = 0 at 12 months | Fisher/Newcombe + logistic marginal effects | Baseline S-FMDRS + disease duration | Exploratory |
| Post-baseline course | S-FMDRS at 6 and 12 months | Linear mixed-effects model | Pathway-period, time, interaction, baseline S-FMDRS, disease duration; participant random intercept | Supportive |
| Attrition sensitivity | Motor symptom-free status at 6 and 12 months | Extreme-case bounds | 6 missing UC + 4 missing NMT-A | Sensitivity |

**Supplementary Results**

**Supplementary Table 4S.** Motor-outcome models and distributional sensitivity analyses.

| **Analysis** | **NMT-A** | **UC** | **Effect estimate (95% CI)** | **P** |
| --- | --- | --- | --- | --- |
| Primary 6-month S-FMDRS (HC3) | Adjusted mean 3.58 (1.84 to 5.32) | Adjusted mean 5.24 (3.59 to 6.90) | Mean difference -1.66 (-4.04 to 0.72) | .166 |
| + functional weakness sensitivity | — | — | Mean difference -1.61 (-4.05 to 0.84) | .192 |
| Log disease-duration sensitivity | — | — | Mean difference -1.35 (-3.94 to 1.25) | .300 |
| BCa bootstrap, 10,000 resamples | — | — | Mean difference -1.66 (-4.25 to 0.45) | — |
| Mann-Whitney sensitivity | Median 0.0 | Median 4.0 | U = 179; rank-biserial r = -0.274 | .115 |
| 6-month motor symptom-free, observed | 16/29 (55.2%) | 1/17 (5.9%) | RD +49.3 pp (21.8 to 66.4); crude OR 19.69 (2.30 to 168.85) | .001 |
| 6-month Firth adjusted | — | — | Adjusted OR 26.49 (4.28 to 326.99) | <.001 |
| 6-month Firth + weakness | — | — | Adjusted OR 20.93 (3.46 to 249.77) | <.001 |
| 12-month motor symptom-free, observed | 25/29 (86.2%) | 9/17 (52.9%) | RD +33.3 pp (6.5 to 56.8); crude OR 5.56 (1.34 to 23.02) | .019 |
| 12-month adjusted logistic | — | — | Adjusted OR 13.3 (1.96 to 89.7) | .008 |
| 12-month average marginal effect | — | — | Adjusted RD +42.1 pp (16.8 to 67.5) | .001 |
| LMM pathway-period effect | — | — | B = -1.228 (-3.122 to 0.666) | .204 |
| LMM time contrast (6 months minus 12 months) | — | — | B = 2.690 (1.724 to 3.655) | <.001 |
| LMM pathway-period x time | — | — | B = -0.621 (-2.552 to 1.311) | .526 |

**Supplementary Table 5S. Attrition analyses.** A. Baseline comparison of analyzed participants and follow-up exclusions. B. Extreme-case sensitivity for 6-month motor symptom-free status. C. Extreme-case sensitivity for 12-month motor symptom-free status. *Note*: Risk differences are calculated as NMT-A minus UC with 95% Newcombe confidence intervals. Extreme-case scenarios assign outcomes only to the 10 participants with unavailable follow-up (6 UC, 4 NMT-A); they are sensitivity bounds rather than imputed estimates.

**A.**

| **Variable** | **Analyzed participants (n=46)** | **Follow-up exclusions (n=10)** | **P** |
| --- | --- | --- | --- |
| Age at diagnostic assessment, mean (SD), years | 14.2 (2.3) | 14.5 (2.0) | .655 |
| Female sex, n (%) | 29 (63.0) | 8 (80.0) | .467 |
| Baseline S-FMDRS, median [IQR] | 10.0 [8.0-12.0] | 5.0 [0-13.5] | .127 |

**B.**

| **Scenario** | **NMT-A** | **UC** | **Risk difference, pp (95% CI)** |
| --- | --- | --- | --- |
| Observed complete cases | 16/29 (55.2%) | 1/17 (5.9%) | +49.3 (21.8 to 66.4) |
| All missing = not motor symptom-free | 16/33 (48.5%) | 1/23 (4.3%) | +44.1 (21.1 to 60.8) |
| All missing = motor symptom-free | 20/33 (60.6%) | 7/23 (30.4%) | +30.2 (3.6 to 51.1) |
| Least favourable to NMT-A | 16/33 (48.5%) | 7/23 (30.4%) | +18.1 (-7.9 to 40.1) |
| Most favourable to NMT-A | 20/33 (60.6%) | 1/23 (4.3%) | +56.3 (32.5 to 71.4) |

**C**.

| **Scenario** | **NMT-A** | **UC** | **Risk difference, pp (95% CI)** |
| --- | --- | --- | --- |
| Observed complete cases | 25/29 (86.2%) | 9/17 (52.9%) | +33.3 (6.5 to 56.8) |
| All missing = not motor symptom-free | 25/33 (75.8%) | 9/23 (39.1%) | +36.6 (10.5 to 57.1) |
| All missing = motor symptom-free | 29/33 (87.9%) | 15/23 (65.2%) | +22.7 (0.6 to 44.3) |
| Least favourable to NMT-A | 25/33 (75.8%) | 15/23 (65.2%) | +10.5 (-12.6 to 33.9) |
| Most favourable to NMT-A | 29/33 (87.9%) | 9/23 (39.1%) | +48.7 (23.6 to 67.2) |

**Supplementary Figure 2S.** Individual S-FMDRS trajectories in the sequential care-pathway cohorts. Raw S-FMDRS scores are shown at baseline, 6 months, and 12 months for all analyzed participants (UC, blue circles, n = 17; NMT-A, orange triangles, n = 29). Lines connect repeated observations from the same participant; points are horizontally jittered to display overlapping observations, particularly at the lower boundary of the scale.
